# Fluorescent Sensing of Chlorophenols in Water Using an Azo Dye Modified β-Cyclodextrin Polymer

**DOI:** 10.3390/s110504598

**Published:** 2011-04-27

**Authors:** Phendukani Ncube, Rui W. Krause, Bhekie B. Mamba

**Affiliations:** Department of Chemical Technology, Faculty of Science, University of Johannesburg, P.O. Box 17011, Doornfontein 2028, South Africa; E-Mails: p.ncube@yahoo.co.uk (P.N.); bmamba@uj.ac.za (B.B.M.)

**Keywords:** fluorescence, chemosensor, chlorinated by-product, azo dye, β-cyclodextrin polymer

## Abstract

A water soluble azo dye modified β-cyclodextrin polymer **4** was synthesized and used as a chemosensor for the detection of chlorinated phenols, model chlorinated by-products (CBPs) of water treatment for drinking purposes. The characterization of the intermediates and the azo dye modified β-CD polymer was done by UV/Vis Spectrophotometry, FT-IR and ^1^H-NMR spectroscopies. The chlorophenols were capable of quenching the fluorescence of the polymer. The polymer showed greater sensitivity towards 2,4-dichlorophenol, with a sensitivity factor of 0.35 compared to 0.05 and 0.12 for phenol and 4-chlorophenol, respectively. The stability constants (K_s_) of the pollutants were also determined by the Benesi-Hildebrand method to be 2.104 × 10^3^ M^−1^ for 2,4-dichlorophenol and 1.120 × 10^2^ M^−1^ for 4-chlorophenol.

## 1. Introduction

**Scheme 1.**
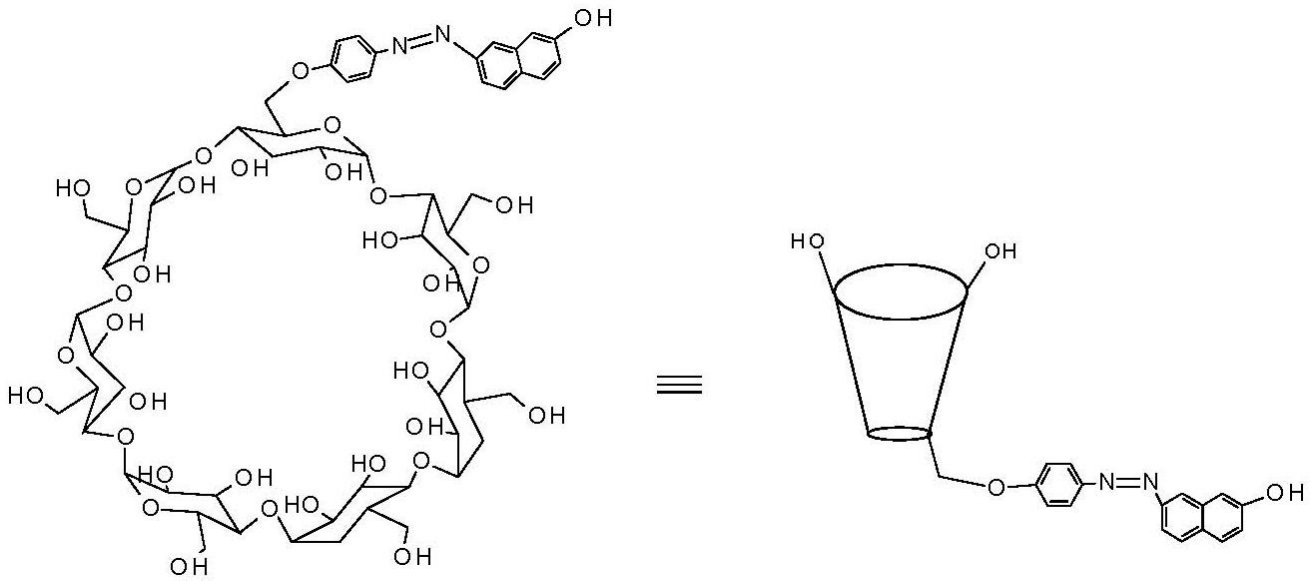
Structure of azo dye modified β-cyclodextrin.

Fluorescent chemosensors based on modified cyclodextrins (CDs) have been of particular interest for a number of reasons. Fluorescence spectroscopy is one of the highly sensitive and selective analytical techniques [1,2]. On the other hand, CDs are able to form host-guest complexes with a wide variety of organic and inorganic compounds by the inclusion phenomena [3–6]. CDs are cyclic oligosaccharides made up of six (α-CD), seven (β-CD) or eight (γ-CD) α-1,4-linked glucopyranose subunits. Generally the most useful of these is β-CD because it is the most easily accessible and the cheapest [3] and it was the one used for this study (Scheme 1).

β-CD forms inclusion complexes with several organic guest molecules, including azo dyes [7], by incorporating the guest molecule into its hydrophobic cavity. Since the electronic environment inside and outside the cavity are different, the behavior of the encapsulated dye is also different in the two environments. This is the principle of many chemosensors based on cyclodextrins. The presence of certain guest molecules can be detected by the changes in the spectroscopic behavior of the attached dye depending on the change in the location of the dye in the presence of the guest.

A number of fluorescent CDs have been synthesized and used as chemosensors for the detection of contaminants in water, including volatile organic compounds (VOCs) [8,9], alcohols [10], amines [11]; and the detection of biological molecules like amino acids [12,13], porphyrin derivatives [14] and bile salts [15]. The fluorophore is often covalently attached to one of the primary carbons on position 6 (C-6) of the cyclodextrin. This attachment is achieved by first substituting the C-6 hydroxyl group with a good leaving group like a tosylate, and then replacing the tosylate with a carboxylate to form an ester linkage [10], or with an amine group to form an amide linkage [15] or with an alcohol to form an ether linkage [9].

Chlorinated by-products (CBPs) are formed in water as a result of the reaction of chlorine and its derivatives, used in the disinfection of water, with natural organic matter (NOM) [16,17]. The trihalomethanes (THM), haloacetonitriles (HAN), halophenols, halogenated aldehydes and ketones and haloacetic acids (HAA) are some of the common CBPs that have been detected in drinking water [16]. These are known to have serious health effects like cancer, liver, kidney, reproductive and developmental effects [16,17], hence the interest in the development of chemosensors for the detection of these pollutants in water.

In this work, therefore, we report on the detection of selected CBPs using a fluorescent azo dye modified β-CD copolymer. The synthesis of the dye modified cyclodextrin (Scheme 1) and of the polymer was carried out using protocol found in literature [18–21] and the characterization done by UV/Vis Spectrophotometry, FT-IR and ^1^H-NMR spectroscopies. The response to the selected CBPs was then done using Fluorescence Spectroscopy.

## 2. Experimental Section

### 2.1. Materials and Methods

#### 2.1.1. Materials

All chemicals, unless otherwise stated, were purchased from Sigma-Aldrich and were used without further purification. Solvents were distilled before use.

#### 2.1.2. Instruments

Absorption spectra were recorded on a Shimadzu UV-2450 UV-VIS Spectrophotometer at room temperature. Fluorescence measurements were done on a Perkin Elmer LS45 Fluorescence Spectrometer whereas Infrared spectra were taken on a Perkin Elmer Spectrum 100 FT-IR Spectrometer in the Attenuated Total Reflectance (ATR) mode. ^1^H-NMR spectra were obtained from a 400 MHz Bruker Avance spectrometer in deuterated water (D_2_O).

### 2.2. Synthesis

#### 2.2.1. Azo Dye Modified β-CD

The azo dye, obtained by diazo coupling of 4-hydroxyaniline with 2-naphthol, was stirred in DMF under a nitrogen atmosphere for one hour. The monotosylated β-CD, obtained by the reaction of β-CD with p-toluenesulphonic anhydride (Ts_2_O) in an aqueous media [18], was then added and the reaction mixture heated at 80 °C under a nitrogen atmosphere for 12 hours. The reaction mixture was then poured into acetone and the resulting precipitate filtered off, washed with copious amounts of acetone and dried to give an orange powder. Yield: 75%. FT-IR: 3292 cm^−1^ (broad) O-H stretch; 1,219 cm^−1^ & 1,025 cm^−1^ (sharp) C–O–C. ^1^H-NMR: Dye protons; d, δ = 7.17–7.19 and d, δ = 7.51–7.53; and s, δ = 7.75.

#### 2.2.2. Polymerization

β-CD (2.515 g, 1.90 mmol) and β-CD-dye (0.253 g, 0.185 mmol) were stirred in DMF (30 mL) under a nitrogen atmosphere for 5 min. Ethylene glycol ditosylate (0.790 g, 1.90 mmol) dissolved in DMF (5 mL) was added dropwise with continued stirring. Three drops of dibutyltin laurate (DBTDL) catalyst were added and the mixture stirred for a further hour. The resulting mixture was added drop wise to acetone (200 mL) and the solid formed filtered off, washed with acetone and dried in a dessicator to give an orange powder (2.340 g, 93% m/m yield of β-CD). FT-IR: 3,331 cm^−1^ (broad) O-H stretch, 1,654 cm^−1^ & 1,081 cm^−1^ C–O–C (sharp). ^1^H-NMR: Dye protons; d, δ = 7.16–7.18 and d, δ = 7.49–7.52; and s, δ = 7.75; CH_2_(O) m 3.38–3.48.

## 3. Results and Discussion

### 3.1. Synthesis

**Scheme 2.**
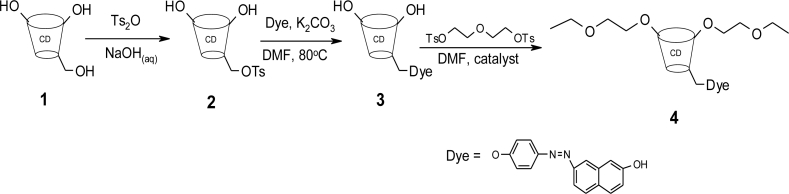
Synthetic route to cyclodextrin polymer **4.**

The dye modified β-CD polymer **4** was synthesized as outlined in Scheme 2 below, based on methods described in the literature [18–22].

Starting from β-CD **1**, a tosyl group was introduced at C-6 by use of Ts_2_O. Previous tosylation of β-CD has been achieved by the use of p-TsCl but this reaction suffers from low yields [23]. An improved method using Ts_2_O under alkaline conditions [18] was used for this synthesis giving an average yield of above 70%. ^1^H-NMR spectroscopy confirmed the presence of aromatic protons (δ = 7 d and δ = 8 d) and the methyl protons at δ = 2. The dye was then attached to the β-CD by replacing the good leaving tosyl group through the phenoxy group of the dye to form the ether linked dye modified β-CD **3**. The UV/Vis spectrum of **3** showed a λ_max_ of 480 nm characteristic of the azo dye, suggesting that the attachment of the dye does not alter its absorption properties. The ^1^H-NMR spectrum of **3** showed no triplet suggesting the structure of the dye modified β-CD as shown in Scheme 2.

On polymerization of the dye modified β-CD, the λ_max_ was again not altered, confirming that the process of polymerization does not change the dye structure. Appearance of a triplet signal at δ = 3 in the ^1^H-NMR spectrum of **4** confirmed the incorporation of the ethylene glycol linker.

**Figure 1.**
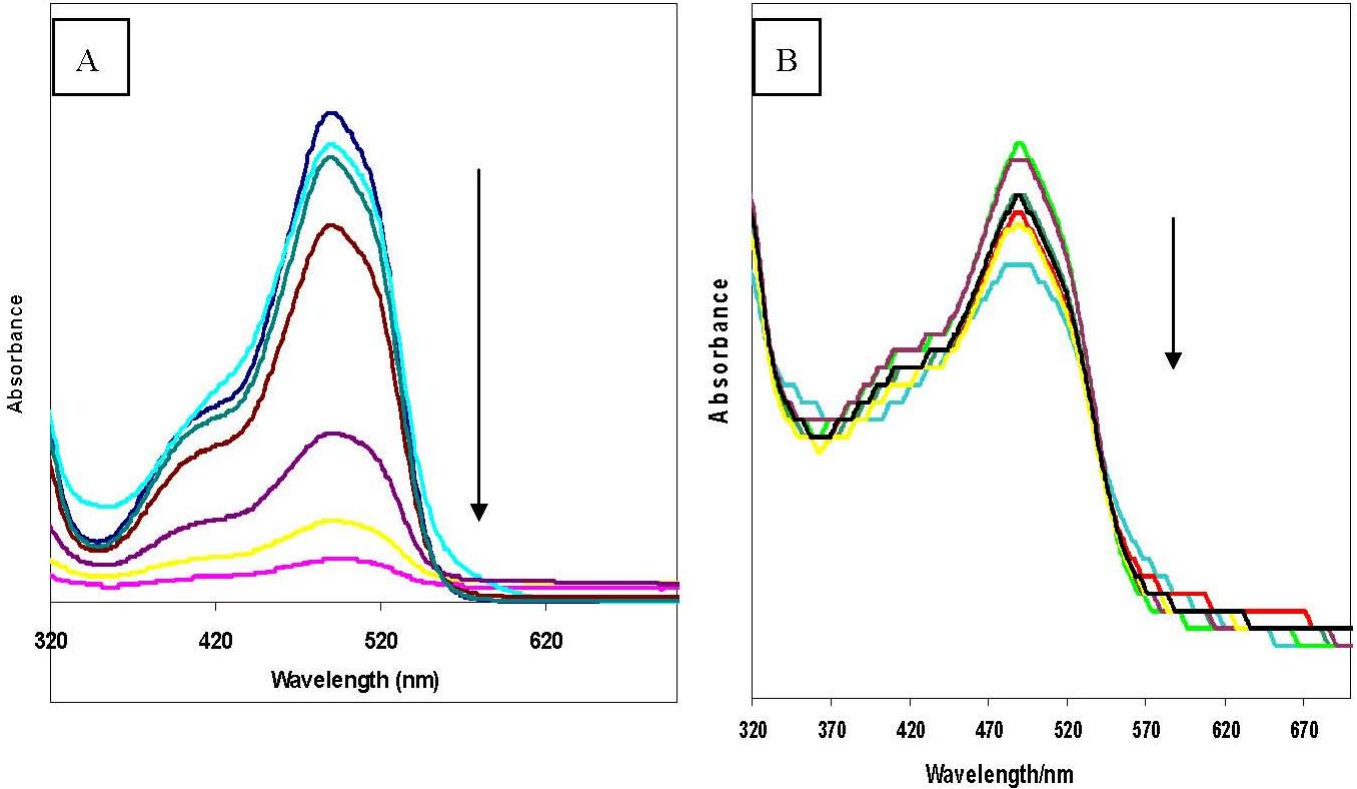
Effect of pH on (**A**) azo dye modified β-CD and (**B**) azo dye modified β-CD polymer.

To have an insight into the conformational structure of the azo dye β-CD polymer, the effect of pH on the absorbance of the dye was investigated. The pH dependence of the absorption of **3** and **4** are shown in Figure 1.

As the pH was increased (pH 6–13) the absorption of **3** was greatly reduced as compared to that of **4**. This suggests that the polymer matrix acts as a form of protection to the dye moiety, making it more stable even in very alkaline conditions. This stability in the polymer matrix is important in investigating the guest induced response properties of the polymer as different solutions may have different pHs.

**Figure 2.**
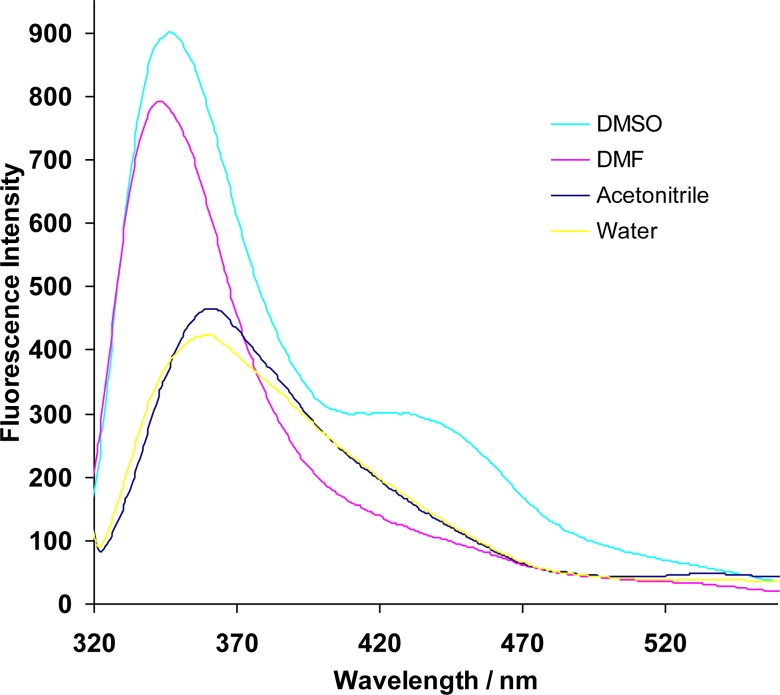
Fluorescence spectra of the azo dye CD polymer in different solvents. (λ_exc_ = 305 nm).

These observations suggest that the dye moiety is encapsulated inside the β-CD cavity at neutral conditions and lies outside the cavity under alkaline conditions. The encapsulation of the dye moiety was also confirmed by investigating the effect of different solvents on the fluorescence of the azo dye β-CD polymer (Figure 2).

The fluorescence intensity of the polymer is greatly enhanced in DMSO and in DMF as compared to acetonitrile and water. A blue shift is also observed with the former solvents with the fluorescence being shifted from around 370 nm for acetonitrile and water to around 350 nm for DMSO and DMF. The polarity of the solvents used is in the order H_2_O > DMSO > CH_3_CN > DMF. Since there is no correlation between the polarity and the observed effect on fluorescence, a possible explanation can come from the fact that both DMF and DMSO have bulkier methyl groups as compared to the other two solvents and therefore similar effects.

### 3.2. Sensing Properties

The response of the azo dye β-CD polymer towards CBPs was investigated by determining the sensitivity of the polymer towards the selected CBPs and binding constants of the sensor/CBP inclusion complexes using fluorescence spectroscopy [8,15].

#### 3.2.1. Sensitivity Factors

**Figure 3.**
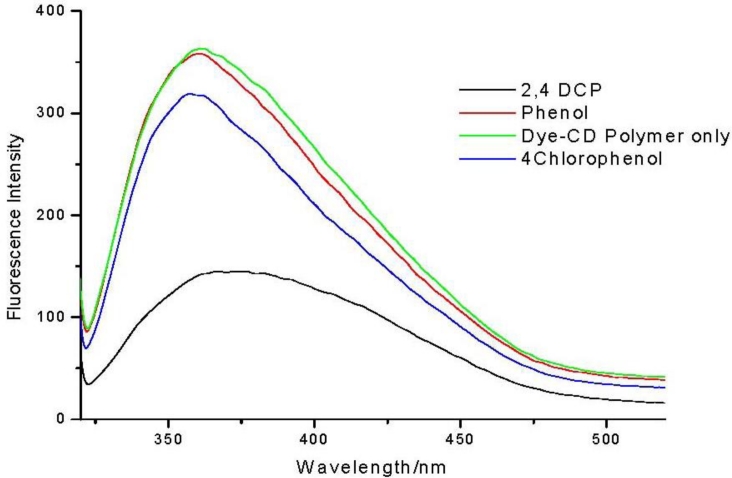
Fluorescence spectra of dye modified CD polymer on addition of different pollutants. {[Dye-CD-Polymer] = 50 ppm; [Pollutant] = 2.0 mM}.

To calculate the sensitivity of the azo dye modified cyclodextrin polymer towards the pollutants, the same amount of pollutant (2 mM) was added to separate aqueous solutions (50 ppm) of the polymer (Figure 3). Fluorescence intensity of the polymer solution was greatly quenched by addition of 2,4-dichlorophenol as compared to phenol and 4-chlorophenol.

The sensitivity towards different pollutants is then estimated by calculating sensitivity factors (SF) as:

$$\text{SF}=\Delta \text{I}/{\text{I}}_{\text{o}}$$

where, ΔI = I_o_ – I and I_o_ is the fluorescence intensity in the absence of pollutant and I is in the presence of pollutant [8]. The sensitivity factors for phenol, 4-chlorophenol and 2,4-dichlorophenol are shown in Table 1.

The sensitivity factors show that the polymer has greater sensitivity towards the chlorinated phenols as compared to phenol. Between the chlorophenols the polymer is more sensitive towards 2,4-dichlorophenol. This result means that the synthesized polymer can be used for molecular recognition of chlorophenols.

**Figure 4.**
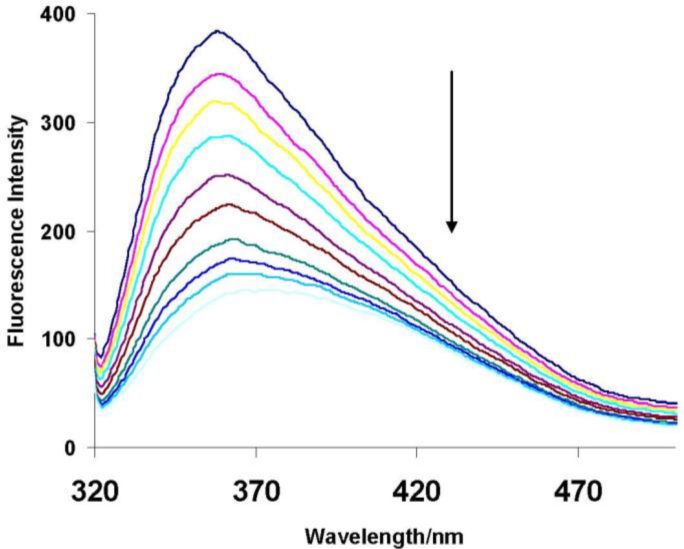
Fluorescence quenching by addition of increasing amounts of 2,4-DCP. {[Dye-CD-Polymer] = 50 ppm; [2,4-DCP] = 0 mM to 2.7 mM}.

To closely investigate the molecular recognition of the pollutants in aqueous solution by the dye modified polymer, titration experiments were carried out. Figure 4 below shows the effect of adding 2,4-dichlorophenol to a 50 ppm aqueous solution of the polymer. On adding small amounts of the pollutant (0–350 μL) fluorescence intensity was quenched, very small volumes of pollutant being added in such experiments to ensure that any observed change is not due to dilution effects [8].

**Figure 5.**
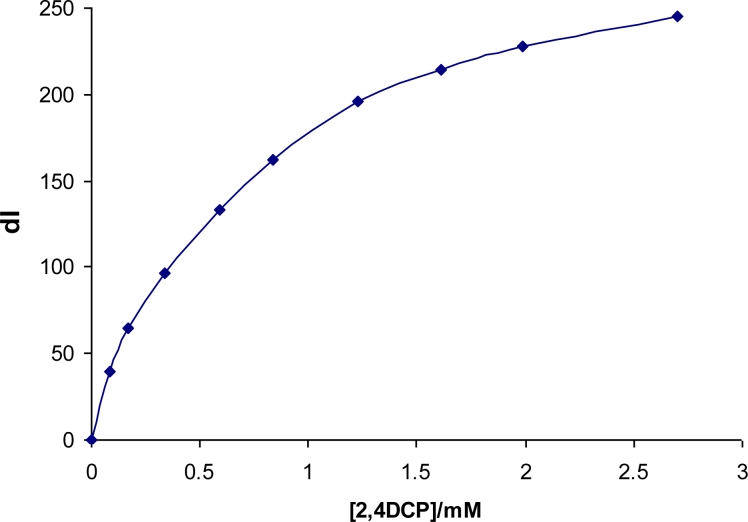
Plot of concentration of 2,4-DCP *versus* the change in fluorescence intensity (ΔI) with each addition of 2,4-DCP.

Figure 5 shows a plot of ΔI *versus* the concentration of pollutant added, where:

$$\Delta \text{I}={\text{I}}_{\text{o}}-\text{I}$$

where, I_o_ is the fluorescence intensity in the absence of pollutant and I is in the presence of pollutant.

As the concentration of the pollutant increases, the change in fluorescence intensity gradually becomes small indicating that the change observed is due to interaction between the cyclodextrin and the pollutant. As the concentration increases the cyclodextrin cavities get saturated and therefore no more change in fluorescence is observed.

In general fluorescence intensity of a substituted cyclodextrin decreases when a guest is added because of competitive inclusion, that is, the preferred binding of the guest into the cyclodextrin cavity excludes the fluorescent substituent out of the cavity into the hydrophilic aqueous environment, thereby quenching its fluorescence [15]. In a similar way, for our case, it can be inferred that the encapsulated azo dye group is excluded from the cyclodextrin cavity on addition of the chlorophenols, into the aqueous environment, thus the observed fluorescence quenching (Figures 3 and 4). The 2,4-dichlorophenol guest displaces most of the azo dye moiety, hence the greater quenching of fluorescence. This implies that the 2,4-dichlorophenol guest complexes most strongly with the cyclodextrin cavity and this is validated by calculating stability constants.

#### 3.2.2. Stability Constants

**Figure 6.**
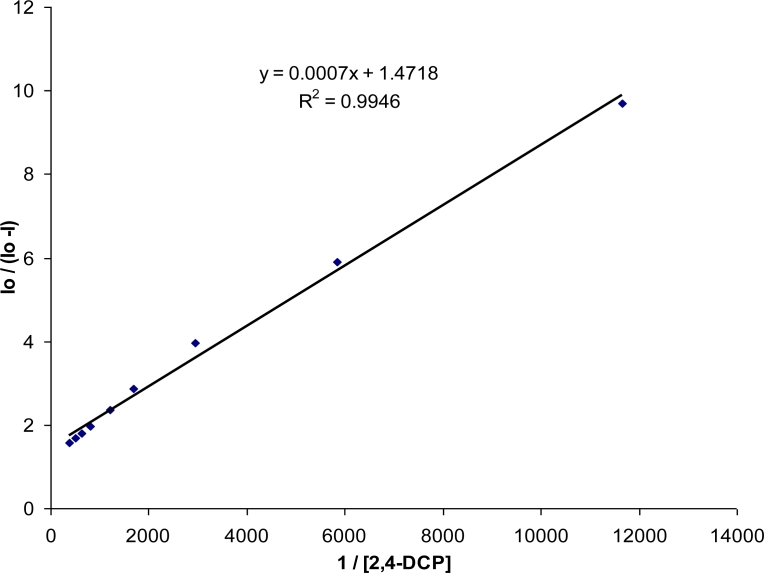
Benesi-Hildebrand plot of CD dye polymer in presence of 2,4-DCP (0–2.7 mM).

Stability constants, K_s_ also referred to as binding constants or association constants were determined from Benesi-Hildebrand plots [24,25]. These plots are linear for a 1:1 stoichiometry. Figure 6 below shows a Benesi-Hildebrand plot for the CD dye polymer in the presence of increasing amounts of 2,4-dichlorophenol.

A very good correlation coefficient of 0.9946 was seen for the plot, showing that the possible complex formed between the cyclodextrin and the pollutant is of a 1:1 stoichiometry. From the plot stability constants (K_s_) were calculated by taking the intercept to slope ratio [25].

$${\text{K}}_{\text{s}}=\text{y-intercept}/\text{slope}$$

which for 2,4-DCP from Figure 6 was found to be 2,104. Similar plots were made for the other pollutants and are summarized in Table 2.

Inclusion complexes formed between β-cyclodextrins and chlorophenols have been studied by several other researchers [26–28]. The general trend seen is that the stability constants increase with the number and position of chloro substituents, which is in agreement with our results. Table 2 also shows stability constants obtained by Leyva *et al.* [26] using different methods. The stability constant obtained with our β-cyclodextrin derivative with 2,4-dichlorophenol is higher than obtained previously while that for 4-chlorophenol is lower. This may be good in the selectivity towards 2,4-dichlorophenol over 4-chlorophenol. The fluorescence difference on adding phenol was so insignificant that it was not possible to calculate stability constants, since the difference fell well into the error of the Fluorescence Spectrometer used (∼ ±20 a.u. fluorescence intensity). However from literature, values of K_s_ for phenol have been calculated to be around 100 using similar β-CD dye derivatives [8]. This means that our β-CD dye polymer forms very stable complexes with 2,4-dichlorophenol (Ks = 2,104) and can be a good probe for the molecular recognition of chlorinated products in water.

## 4. Conclusions

An azo dye modified β-cyclodextrin polymer was synthesized and characterized. The fluorescence of the polymer was quenched by the addition of the chlorophenols studied with sensitivity towards 2,4-dichlorophenol being the greatest among the studied phenols. Stability constants calculated indicated that the inclusion complex formed with this pollutant is the most stable. Such a polymer has the potential to be used as a probe for the molecular recognition of guests in chemosensors for the detection of chlorinated by products in drinking water.

## Figures and Tables

**Table 1.** Sensitivity factors.

**Pollutant**	**Sensitivity Factor**
Phenol	0.05
4-Chlorophenol	0.12
2,4-Dichlorophenol	0.35

**Table 2.** Stability constants.

**Pollutant**	**K_s_ (Fluorescence)**	**Literature K values [26]**	
		**K_s_(UV-Vis)**	**K_s_(^1^H-NMR)**
4-Chlorophenol	112	427	420
2,4-Dichlorophenol	2,104	350	556

* K_s_ value could not be calculated as the fluorescence quenching was very small on addition of phenol.
